# Prolonged Effect of Seminal Plasma on Global Gene Expression in Porcine Endometrium

**DOI:** 10.3390/genes11111302

**Published:** 2020-11-03

**Authors:** Marek Bogacki, Beenu Moza Jalali, Anna Wieckowska, Monika M. Kaczmarek

**Affiliations:** Institute of Animal Reproduction and Food Research of Polish Academy of Sciences in Olsztyn, 10-748 Olsztyn, Poland; beenu.jalali@pan.olsztyn.pl (B.M.J.); anna.kitewska@gmail.com (A.W.); m.kaczmarek@pan.olsztyn.pl (M.M.K.)

**Keywords:** seminal plasma, endometrium, global gene expression, microarray, pig

## Abstract

Seminal plasma (SP) deposited in the porcine uterine tract at the time of mating is known to elicit an initial response that is beneficial for pregnancy outcome. However, whether SP has any long-term effect on alterations in endometrial molecular and cellular processes is not known. In this study, using microarray analyses, differential changes in endometrial transcriptome were evaluated after Day 6 of SP-infusion (6DPI) or Day 6 of pregnancy as compared to corresponding day of estrous cycle. Both, pregnancy and SP induced significant changes in the endometrial transcriptome and most of these changes were specific for a particular group. Functional analysis of differentially expressed genes (DEGs) using Ingenuity Pathway Analysis revealed that inhibition in immune response was affected by both pregnancy and SP infusion. Long-term effects of SP included differential expression of genes involved in inhibition of apoptosis, production of reactive oxygen species and steroid biosynthesis, and activation of processes such as proliferation of connective tissue cells and microvascular endothelial cells. Moreover, interleukin-2 and interferon-γ was identified to be responsible for regulating expression of many DEGs identified on 6DPI. The present study provides evidence for the long-term effects of SP on porcine endometrium that can be beneficial for pregnancy success.

## 1. Introduction

The high rate of pregnancy failure in human and livestock has been attributed mainly to the unsynchronized development of the embryos with the proper preparation of the female reproductive tract and the impaired communication between the developing embryos and uterus [1,2,3].

Understanding of the molecular embryo-maternal cross-talk is crucial for solving infertility problems, reducing pregnancy loss and identifying hormonal, paracrine, and autocrine factors regulating the developmental potential of the offspring. Effective recognition of the embryo in the maternal tract is crucial for the preparation of an appropriate environment in the uterus for the embryo’s development, implantation, and final establishment of pregnancy [4]. However, exactly when the oviduct and uterus recognize the presence of embryos and how the maternal pathway changes its environment in response to embryos is not fully understood.

In pigs, transcriptomic profiling of pregnant and non-pregnant animals has been conducted and pointed major differences in endometrial genes activities in the post-conception period of pregnancy [5], pre-attachment phase [6,7] and during onset of implantation [8]. Identified alterations in uterine transcriptome lead to morphological, biochemical and immunological changes and are reflection of action of para- and autocrine signals released by maternal tract as well as developing embryos. 

The application of artificial insemination (AI) and other reproductive technologies shows that pregnancy can be maintained without any semen being deposited in the uterus (embryo transfer outcome) or with highly diluted semen for AI [9]. However, studies conducted in pigs and other livestock species show reduction of early fetal loss due to exposure to seminal constituents. Seminal plasma (SP) is a mixture of various components and serves not only as a vehicle for sperm transport, protection, and nutrition but also affects gamete interaction and successful establishment of pregnancy. Biologically active molecules present in SP (estrogens, testosterone, prostaglandins, cytokines, and growth factors) interact with uterine epithelium to induce a series of changes in the maternal immune response and modify cellular composition, structure, and function of the reproductive tract, directly in local tissues or indirectly in tissues distal to the uterus, for example ovary [10]. In pigs, it was shown that introduction of SP before natural service or/and AI leads to the increase in farrowing rate and litter size [11]. Additionally, increased litter size was also reported after pre-sensitization of the uterus to sperm and seminal antigens in a previous estrous cycle [12] and an increased embryo survival was noted after addiction of leukocyte antigens to boar semen at breeding [13]. 

The immediate response to full semen insemination in pigs is a rapid influx of inflammatory cells such as neutrophils into the uterine lumen [14] and macrophages, granulocytes, and lymphocytes into the endometrial stroma [15]. The activation of maternal immune system does not cause the rejection of seminal antigens due to the presence of several immunoregulatory molecules in boar SP, such as prostaglandin E (PGE) and tumor growth factor β (TGF-β) [16]. It has been suggested that constituents of SP deposited in lower reproductive tract can easily access the upper reproductive tract and induce biologically relevant changes in the endometrium [17]. Concomitant with this observation, SP interacts with uterine cells in pigs to induce expression of several cytokines: granulocyte-macrophage colony-stimulating factor (GM-CSF), interleukin 6 (IL-6), and macrophage chemotactic protein-1 (MCP1) 34 h after infusion [18]. Moreover, components of SP can alter expression of prostaglandin synthesis enzymes in the porcine oviduct and uterus, acting in favor of PGE2 action as a critical element of early embryo transport and development but also can modify endometrial vascularity up to 10 days after infusion [19,20]. Additionally, in our previous study, prolonged modulatory effects of SP infusion at least for 6 days, demonstrated by induction of T helper (Th) and T regulatory (Treg) cells, increased interleukin 10 (IL10), and decreased expression granulocyte-macrophage colony stimulating factor (GM-CSF), was observed [21]. 

Though, the immune modulatory effects of SP on porcine endometrium are well documented, to our knowledge there is no published microarray data showing long term effect of SP on molecular changes in porcine endometrium that might be relevant for hatching blastocysts on Day 6 of pregnancy when they reach the uterine horn from the oviduct. That is why, we hypothesized that SP infusion can induce not only immediate but also prolonged transcriptome changes required for endometrial receptivity and modulation of immune response in the uterine environment.

## 2. Materials and Methods 

### 2.1. Animals and Treatments

All procedures involving the use of animals were conducted in accordance with the national guidelines for agricultural animal care and approved by the Animal Ethics Committee, University of Warmia and Mazury in Olsztyn, Poland; Decision 86/2011. Estrous induction and synchronization, insemination, and SP infusion were performed as previously described [21]. Cross-bred gilts (*Sus scrofa domesticus*) of similar age and weight were subjected to a hormonal treatment with an intramuscular injection of 750 I.U equine chorionic gonadotropin (eCG) and 500 I.U. human chorionic gonadotropin (hCG) given after 72 h. Gilts randomly divided into three groups (*n* = 5), 24 h after hCG injection were treated as following: (1) artificially inseminated twice within an interval of 12 h, (2) received two intrauterine infusions of 100 mL SP with an interval of 12 h or (3) received two intrauterine infusions of 100 mL PBS within 12 h (reference group). All the treatments were given at two time points within an interval of 12 h to mimic regular procedure of artificial insemination. Artificial insemination was performed with 100 mL of 2.5 × 109 spermatozoa diluted from the fresh semen collected from a boar with semen extender Safe Cell + (IMV technologies, L’Aigle, France). All the treatments were deposited into the uterus using post cervical artificial insemination methods (PC Blue, SafeBlue Foamtip^®^ with PC Cannula-Minitube) to facilitate interaction of treatments with the endometrium. SP for intrauterine infusion was prepared from whole semen collected and pooled together from four fertile boars, which were used for AI. SP was separated by centrifugation of the whole semen at 1200× *g* at 4 °C for 20 min, divided into 100 mL aliquots and frozen at –20 °C until needed for intrauterine infusion. Animals were slaughtered in a local abattoir at day 6 of pregnancy or at day 6 after SP or PBS infusion. Uterine horns were flushed with PBS and opened longitudinally along the anti-mesometrial surface. Endometrial explants were collected from the upper part of uterine horns and snap-frozen in liquid nitrogen. In the group of artificially inseminated animals, only those animals were included in the study in which pregnancy was confirmed by the presence of blastocysts in the uterine flushings. 

### 2.2. RNA Isolation

Total RNA was isolated from 30 mg of grinded in liquid nitrogen and homogenized endometrial tissue with the use of a Qiagen RNeasy Mini Kit (Qiagen, Valencia, CA, USA) and genomic DNA contamination was removed by DNAse treatment (RNase free DNAse Kit, Qiagen, Valencia, CA, USA). The initial RNA quality and quantity were determined spectrophotometry using NanoDrop ND-1000 (Thermo Scientific, Pittsburgh, PA, USA). Subsequently, RNA integrity was evaluated with microfluidic electrophoresis by 2100 Bioanalyzer (Agilent Technologies, Santa Clara, CA, USA) and RNA integrity number (RIN) was calculated for each sample using Agilent 2100 Expert software (Agilent Technologies, Inc., Santa Clara, CA, USA). Only samples with a RIN above 8.0 were processed further.

### 2.3. Microarrays

The Porcine (V2) Gene Expression Microarray 4_44 (Agilent Technologies, Santa Clara, CA, USA) was used for differential gene expression analysis. As positive internal controls of microarrays performance an RNA Agilent Spike-In Kit, One Color was used (Agilent Technologies, Santa Clara, CA, USA). Total RNA obtained from cyclic and pregnant gilts was amplified and labeled with Cy3 dye using Quick Amp Kit, One Color (Agilent Technologies, Santa Clara, CA, USA). After purification of labeled RNA (Qiagen RNeasy Kit), the yield (ng of cRNA) and specific activity (pmol of Cy3/mg of cRNA) were quantified using NanoDrop ND-1000. Labeled cRNA was fragmented, mixed with hybridization buffer, and added to the microarray slide. On each array (*n* = 4, one slide) a combination of samples from all three groups were hybridized for 17 h at 65 °C in an Agilent hybridization oven. Afterwards, arrays were dissociated from the hybridization chamber and washed twice in GE wash buffers. After washing, slides were scanned using Agilent G2565CA Microarray Scanner at settings recommended for the 4_44 K array format. Images obtained after scanning were analyzed using Agilent Feature Extraction software v. 10.5.1.1 (Agilent Technologies Inc., Santa Clara, CA, USA). A detailed analysis including filtering of outlier spots, background subtraction from features, and dye normalization was performed.

### 2.4. Data Analysis

Data obtained after extraction was further analyzed using GeneSpring GX 11.0.2 (Agilent Technologies, Santa Clara, CA, USA). To determine differentially expressed genes (DEGs) data were normalized with quantile method and afterwards moderated *t*-test (Benjamini–Hochberg false discovery rate (FDR) < 0.05, absolute fold change |Fc| > 1.5) was performed to compare endometrial transcriptomes between: (1) pregnant and cyclic (*n* = 5) as well as (2) SP infused and cyclic animals (*n* = 4, data from one array were not correlating with other arrays after principal component analysis (PCA)). For identification of differentially expressed probe sets with unknown target sequence the annotation was done manually using NCBI blast algorithm [22]. When porcine sequence for particular mRNA was not available the annotation was performed for human, murine, and cattle transcript, and the date was included only if the query cover and percent identity was equal or higher than 70%. To identify biological processes, pathways and upstream regulators Ingenuity Pathway Analysis (IP Ingenuity Systems-Qiagen, Aarhus, Denmark). Biological processes, pathways, and upstream regulators were considered statistically significant if Fishers exact tests *p*-value ≥ 0.05 and each process associated with at least four DEGs. Biological processes and pathways connected to cancer, diseases and disorders, nervous, respiratory, digestive, renal and urological system, organismal survival and functions, drug metabolism, organ development and behavior were not taken into consideration while examining IPA results. 

### 2.5. Quantitative Real-Time PCR

For validation of microarray results 2 µg of total RNA were transcribed to cDNA with the use of High Capacity RNA to cDNA kit (Applied Biosystems, Foster City, CA, USA). Real-time PCRs were performed using 7900 HT Real-Time PCR System (Applied Biosystems) using 15 ng cDNA, TaqMan Universal MasterMix II, no UNG. TaqMan assays are listed in Table 1. The initial denaturation was carried out at 95 °C 

For 15 min and was followed by 40 cycles of denaturation at 95 °C (15 s) and primer annealing and elongation at 60 °C. Non template controls and non-enzyme controls were included in the experiment. Gene expression levels were calculated with the use of Real-Time PCR Miner (http://ewindup.info/miner/) and normalized using the geometric mean of expression levels of two reference genes-hypoxanthine guanine phosphoribosyl transferase (HPRT) and β-actin (ACTB). The statistical differences in gene expression between the endometrium from pregnant and cyclic as well as seminal plasma infused and cyclic animals was analyzed with GraphPad PRISM v. 5.0 Software (GraphPad Software, Inc., San Diego, CA, USA) by Student’s *t*-test. Confirmed differences in gene expression (*p* < 0.05) were expressed as fold changes.

## 3. Results

### 3.1. Differential Changes in Endometrial Transcriptome

Pairwise comparisons of endometrial samples collected from pigs on Day 6 of pregnancy (6DP) and Day 6 after SP infusion (6DPI) with PBS-infused cycling control pigs on corresponding day of estrous cycle (6DC) were performed to identify endometrial transcriptome changes in response to pregnancy and SP constituents, respectively. Statistical analysis revealed a pregnancy-induced change (fold change > 1.5 or < −1.5; *p* < 0.05; false discovery rate (FDR) = 5%) in 444 probes representing 281 differentially regulated (DEGs) of which 225 were downregulated and 56 were upregulated, on Day 6 as compared to controls on 6DC (Table S1). Whereas, genes showing the most downregulation included *S1008*, *CGA*, and *HLA-DQA1*, genes with highest upregulation were *GPR116*, *COL4A1*, and *CADPS2*. On the other hand, SP-infusion resulted in statistically significant alterations in 342 probes representing 255 DEGs. A downregulation of 118 genes and upregulation of 137 genes was observed on Day 6 after SP infusion as compared to 6DC (Table S2). SP infusion decreased the expression of *ATP6V1C2, NMU, S100A8, S100A12, ANGPTL3, NOS1, CCR3* and upregulated *PCDHB15*, *KLHL5*, *RASGEF1A, NMB*, and *CAPZB*.

### 3.2. Comparison between Pregnancy-Induced and Seminal Plasma-Induced Changes in Endometrial Transcriptome

**Figure 1:**
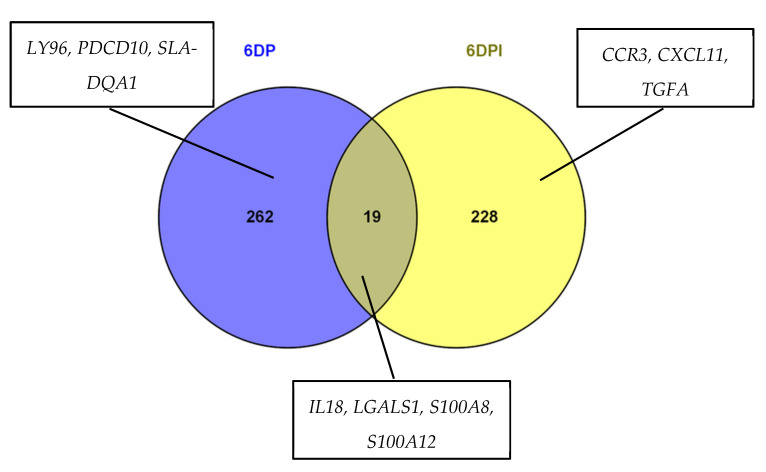
Venn diagram showing number of shared and unique DEGs altered on Day 6 of pregnancy (6DP) and Day 6 after SP infusion (6DPI) in comparison to 6 Day of estrous cycle. Genes chosen for qPCR validation are listed in boxes.

The list of pregnancy and SP-induced DEGs were uploaded to jvenn software (http://jvenn.toulouse.inra.fr/app/example.html) to visualize common DEGs across both presented comparisons and DEGs that were identified as a result of either pregnancy or SP-infusion (Figure 1). This comparison identified 19 common genes (Table S3) that were differentially regulated by pregnancy and SP-infusion. Whereas, in both the groups, 15 genes were found to be downregulated only three genes were upregulated. The expression of transcript coding for chloride channel, voltage sensitive 5 (CLCN5) was lower during Day 6 of pregnancy (Fc = −2.66) and higher on Day 6 after SP infusion (Fc = 1.53). Most of the DEGs common between two groups were involved with immune regulation (*IL15, IL18, LGALS1, FKBP3*) or were DEGs related with molecular transport (*S100A8, S100A12, CLCN5*) and structural organization (*FN1, COL7A1, TUBA1B*).

### 3.3. Analysis of Biological Processes, Pathways, and Upstream Regulators of Identified DEGs

**Figure 2:**
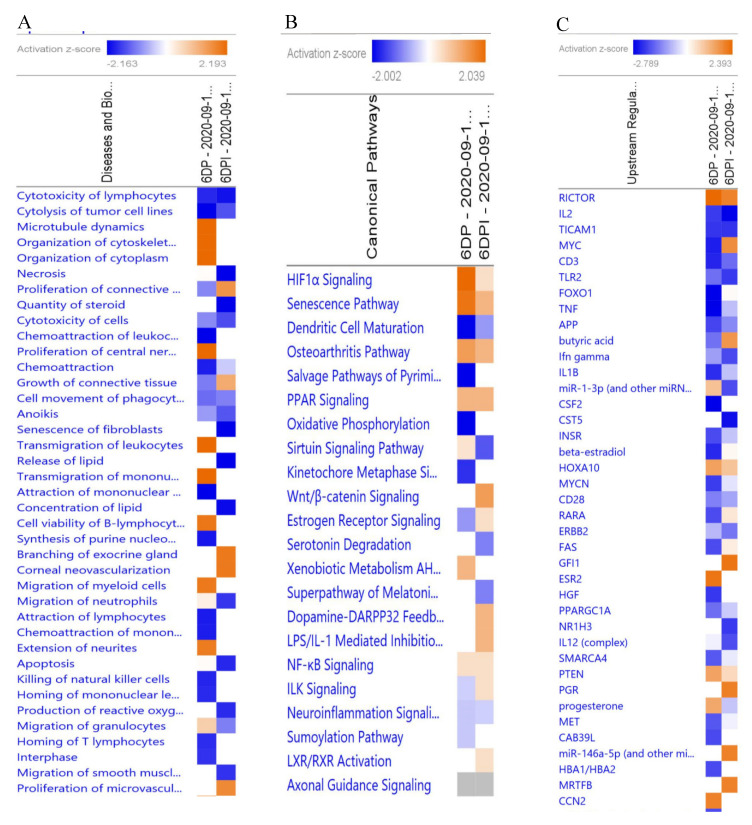
Comparison analysis of (**A**) diseases and bio functions, (**B**) canonical pathways, and (**C**) upstream regulators associated with DEGs identified on 6DP and 6DPI. A *Z*-score > 2 (orange color) is associated with activated functions, pathways, or upstream regulators and a *Z*-score < 2 (blue color) is associated with inhibited functions, pathways, or upstream regulators.

**Figure 3:**
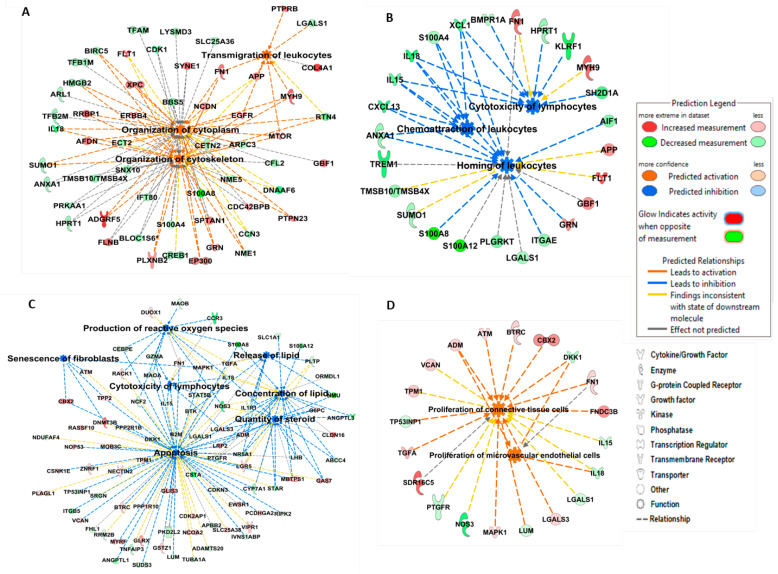
IPA depicting networks integrating DEGs identified on 6DP as compared to 6DC in (**A**) activated functions: organization of cytoskeleton, organization of cytoplasm, and transmigration of leukocytes, and (**B**) inhibited immunological functions: cytotoxicity of lymphocytes, chemoattraction of leukocytes, and homing of leukocytes. DEGs identified that 6DPI as compared to 6DC were found to be associated with (**C**) activated functions: proliferation of connective tissue cells and proliferation of microvascular endothelial cells and (**D**) inhibited functions: cytotoxicity of lymphocytes, apoptosis, production of reactive oxygen species, concentration and release of lipid and quantity of steroid, on Day 6 after SP infusion in the porcine endometrium. Red and green colors depict an increase or decrease, respectively, in the expression of a gene. The color intensity of nodes indicates a fold change increase or decrease associated with a particular DEG.

To classify identified DEGs altered on Day 6 of pregnancy and Day 6 after SP infusion under functional categories, tools available in IPA database were employed. Analysis of DEGs using core analysis module of IPA revealed many altered functions in porcine endometrium as a result of pregnancy or SP-infusion. Furthermore, a comparison analysis, comparing disease and biological functions, canonical pathways, and upstream regulators of DEGs was also performed to evaluate the differences in activated and inhibited functions between pregnant and SP-infused animals (Figure 2). Whereas, most of the identified functions were specific to either pregnancy status or SP-infusion, inhibition of immune functions was common to both the groups. A pregnancy specific activation was observed in processes related to organization of cytoskeleton, organization of cytoplasm, and transmigration of leukocytes (*Z* > 2.0; Figure 3A). The molecular functions inhibited by pregnancy included chemoattraction of leukocytes, homing of leukocytes, and cytotoxicity of lymphocytes (*Z* < −2.0) (Figure 3B, Table S4). On the other hand, SP infusion, besides inhibiting cytotoxicity of lymphocytes, also inhibited molecular functions such as apoptosis, lipid metabolism, senescence of fibroblasts, and production of reactive oxygen species (*Z* < −2.0; Figure 3C, Table S5) and activated only function related to proliferation of microvascular endothelial cells and connective tissue cells (*Z* > 2.0; Figure 3D). Interestingly, alterations in molecular signaling pathways associated HIF1α signaling were specific to Day 6 of pregnancy and activation of Wnt/β-catenin signaling was specific to SP-infused group. Genes associated with PPAR signaling were differentially altered in both the groups (Figure 2B). 

**Figure 4:**
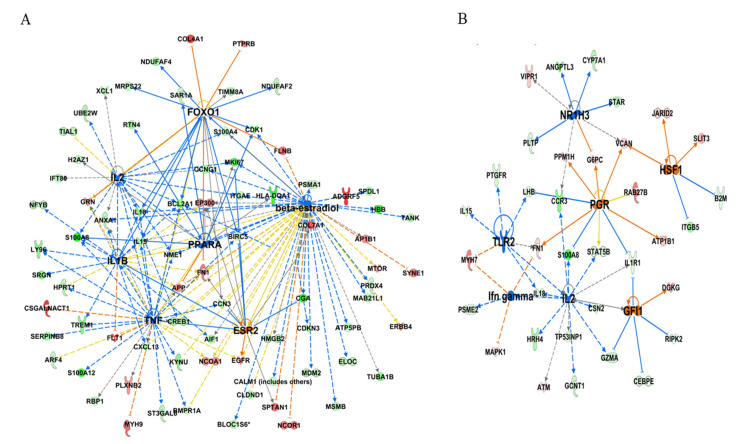
Networks generated for identified upstream regulators of DEGs in porcine endometrium on (**A**) Day 6 of pregnancy or (**B**) Day 6 after SP infusion. Red and green colors depict an increase or decrease, respectively, in the expression of a gene. The color intensity of nodes indicates a fold change increase or decrease associated with a particular DEG.

In our study, upstream analysis function of IPA was used to identify the molecules including cytokines, transcription factors, or hormones (2.0 < *Z* score < −2.0) that might be upstream regulators of altered gene expression in porcine endometrium as a result of pregnancy or SP-infusion. Many cytokines including interleukin (IL-1β), IL-2, TNF, transcription regulators such as FOXO1, NUPR1 and nuclear receptor ESR2, PPARA, and AHR were found to affect the gene expression in endometrium on Day 6 of pregnancy (Figure 4A, Table S6). Upstream analysis of SP-induced DEGs revealed that upstream regulators such as IL-2 and RICTOR were the only common regulators among two groups. Other molecules affecting the expression of genes in SP-infused endometrium on Day 6 included *IFNγ, GFI1, HSF1, TNFRSF1A, TLR2, and NR1H3* (Figure 4B, Table S7). Interestingly, we observed PGR to be an upstream regulator of SP-induced DEGs (Figure 4B).

### 3.4. qRT-PCR Validation of Microarray Results

**Figure 5:**
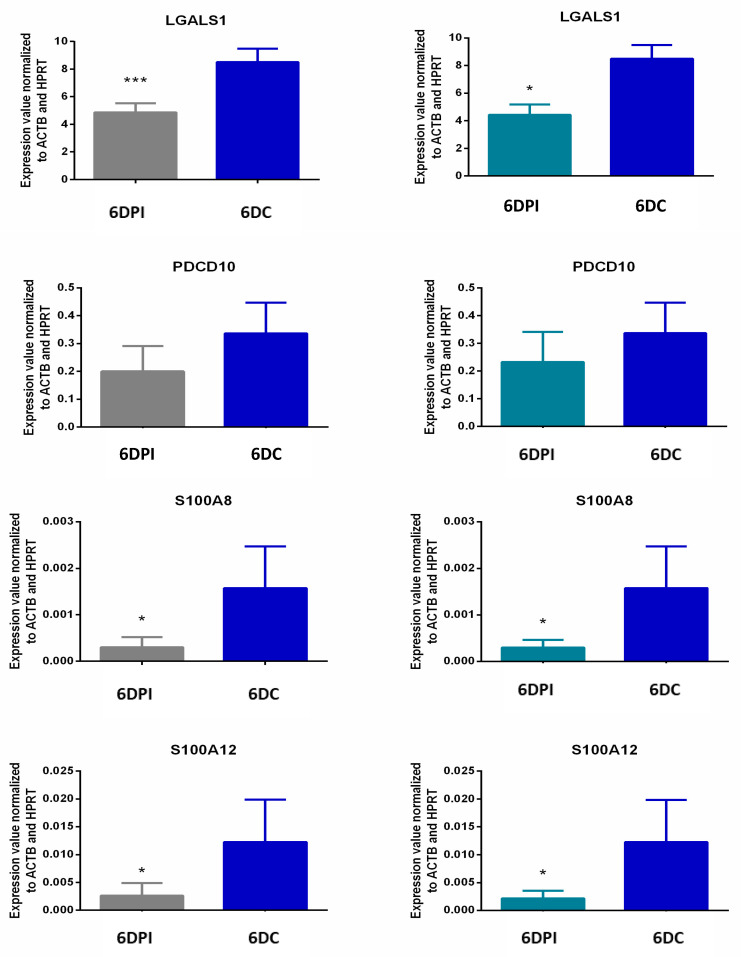

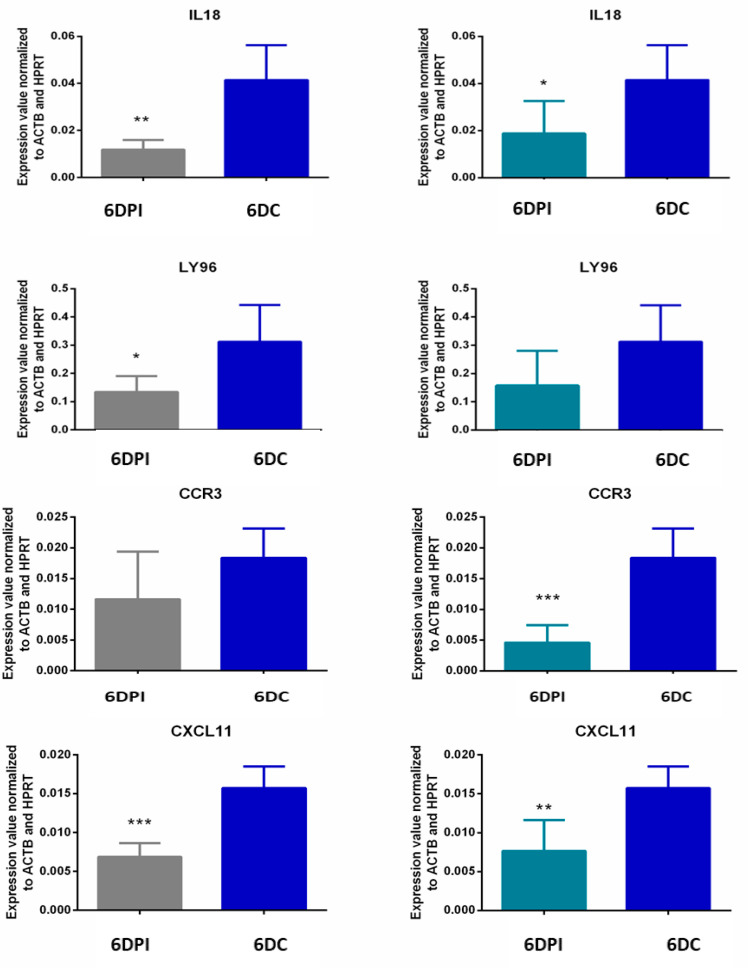

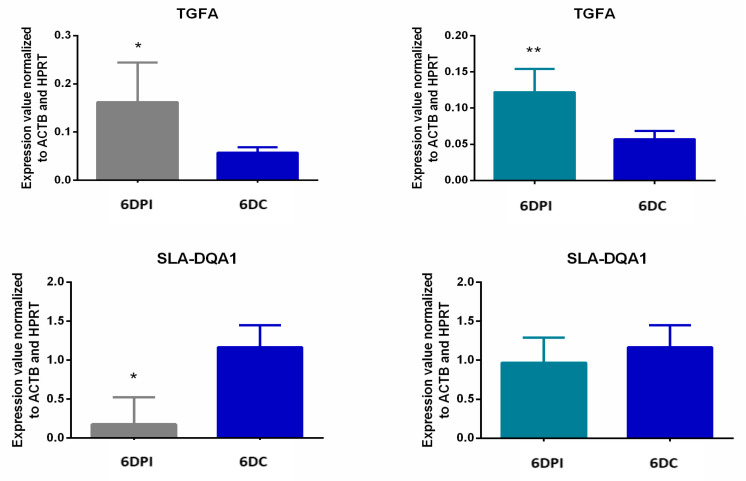
Validation of microarray results using qPCR. Expression of *CCR3* (*chemokine* (*C-C motif*) *receptor 3*), *CXCL11* (*chemokine* (*C-X-C motif*) *ligand 11*), *TGFA* (*transforming growth factor, α*), *LGALS1* (*galectin 1*), *IL-18* (*interleukin 18*), *LY96* (*lymphocyte antigen 96*), *PDCD10* (*programmed cell death 10*), *SLA-DQA1* (*MHC class II histocompatibility antigen SLA-DQA*), *S100A8* (*S100 calcium binding protein A8*), and *S100A12* (*S100 calcium binding protein A12*) in the Day 6 of pregnant or SP infused animals as compared to Day 6 of cycling control animals. Expression values were normalized to expression of *ACTB (Actin, β)* and *HPRT (Hypoxanthine phosphoribosyltransferase1)*. Data are presented as mean ± standard error. * *p* ≤ 0.05, ** *p* ≤ 0.01, *** *p* ≤ 0.001 by *t*-test. The fold change and *p* values are shown in Table 2.

For q PCR validation of microarray data, 10 DEGs, shown in Figure 1, were chosen. These genes were associated with immune function, molecular transport, and cell proliferation. Most of the assessed DEGs showed similar expression profiles when comparing microarray and qPCR data (Figure 5). However, qPCR data revealed a pregnancy induced upregulation of TGFA expression that was not observed in the microarray data. A comparison of fold change and *p* value of DEGs obtained after qPCR and microarray data analysis is presented in Table 2.

## 4. Discussion

Although, nowadays, pregnancy in pigs is a result of AI with diluted semen or the result of embryo transfer techniques during which only the residual SP enters the reproductive tract, there is documented evidence that SP affects the biological functions of the uterus and evidence that interaction between male SP and female tissues promotes fertility, pregnancy, and finally health of offspring [23]. Many transcriptomic studies in humans, cattle, and pigs have been carried out to evaluate the effects of SP on endometrium [17,24,25]. These reports support the results that SP itself modifies the transcriptome, although semen either after mating or AI results in the maximum changes in the molecular profiles in peri-ovulatory uterine tract of pigs [25,26]. However, in pigs, these studies either reported the immediate effects (after 24 h) of SP on uterine tract or effect of SP followed by AI [27]. Our previous studies have shown that SP can induce long term changes in the endometrium that can be observed at least till Day 6 of its infusion, therefore, in this study, we evaluated SP-induced long-term changes in endometrial transcriptome to identify significantly altered molecular and cellular processes that might prepare endometrium for a possible pregnancy. We also compared these changes with the list of DEGs obtained on Day 6 of pregnancy to evaluate distinct and shared pathways between the two treatments.

Our data demonstrated that as many as 255 and 281 genes are differentially regulated after 6 days of SP infusion and on Day 6 of pregnancy as compared to corresponding day of estrous cycle with only 19 being common to both the groups. A comparison of the biological, molecular, and cellular functions altered by SP-infusion or pregnancy revealed that most of these processes are specific to either SP-infused or pregnant groups of animals, highlighting specific actions of SP constituents. Many DEGs found in both the groups were responsible for inhibition of immune function. Processes such as organization of cytoskeleton and transmigration of leukocytes were specific for pregnancy induced DEGs. Treatment with SP inhibited processes such as apoptosis, necrosis, production of reactive oxygen species and steroid transport. On the other hand, connective tissue cell and microvascular endothelial cell proliferation was activated by SP. Whereas, pathways affected by SP, such as endometrial immune response and steroid biosynthesis were inhibited after Day 6 of its infusion, these responses were activated immediately after SP infusion [25].

### 4.1. Immune Regulation

Consistent with the literature reports, modulation of immune responses was one of the topmost processes altered by both the treatments, i.e., AI and SP. Blastocysts and SP are known to differentially regulate genes involved in immune response on Day 6 [5,21]. It is well known that immediate effects of SP on endometrium include an inflammatory reaction, a response to paternal antigens and mainly to clear reproductive tract of any pathogen deposited at the time of mating [13]. A recent study reported minimal effect of SP treatments on the differential expression of genes in the porcine upper reproductive tract [25]. Consistent with these reports, a very small number of immune related genes were differentially regulated in our study. However, there was no difference between the genes regulated either as a result of pregnancy after AI (six DEGs) or SP treatment (eight DEGs) showing the similarities between both the treatments. In the present study genes involved with immune functioning such as *IL15*, *IL18*, and *LGALS1* were found to be downregulated in both the groups and additionally, *STAT5* and *GZMA* was downregulated by SP infusion. Interleukins 15 and 18 are pro-inflammatory cytokines, these cytokines are not only the regulators of innate immune response but also enhance the cytotoxicity of natural killer cells (NK cells) [28,29]. Furthermore, a decrease in cytotoxicity of lymphocytes was also evident from the downregulation of *GZMA*, a factor secreted by the cytotoxic T cells and natural killer cells that induces apoptosis [30]. A downregulation of these factors might result in a dampened innate immune response at the time of blastocyst hatching and in turn may affect endometrial immune tolerance to paternal antigens. Downregulation of many of the genes associated with immune regulation in SP-infused animals was found to be a result of inhibition of toll-like receptor (TLR) 2 and IL-2 signaling. The activation of TLR-signaling is indeed found to be detrimental to the success of pregnancy [5]. In the endometrium of pregnant pigs, negative regulation of immune responses could be a result of inhibition TNF or IL-1 signaling (*Z* > –2.0). Both SP and pregnancy status are able to generate moderate changes in endometrial immune response. Current data closely corresponded with our previous results suggesting that the immunomodulatory effects of SP, also observed at the protein level, last up to at least 6 days after its infusion at which time blastocysts enter the uterus from the oviduct [21,23]. 

### 4.2. Cell Death and Survival

A large number of endometrial genes associated with the category cell death and survival were downregulated in SP-infused animals as compared to PBS-infused controls. These DEGs resulted in an inhibition of the apoptotic signaling and promotion of microvascular and connective tissue cell proliferation. Corresponding to the proliferation of connective tissue, an inhibition in senescence of fibroblasts was also observed, confirming an earlier observation that SP constituents can also have an effect on stromal layer of endometrium [17]. Our data emphasize the utility of SP in suppressing apoptosis in endometrial cells during early pregnancy. Increase in proliferation and inhibition of apoptosis of endometrial cells during early pregnancy period is an important step for generation of receptivity to implanting embryos in pigs [31]. A recent report reveals pro-survival effect of SP on endometrial epithelial and stromal cells [17]. In vitro treatment of human endometrial cells resulted in inhibition of pathways promoting apoptosis [17]. In this study we observed downregulation of many apoptotic genes such as *TNFAIP3, CXCL11, ABCC4,* and *B2M* and upregulation of genes inhibiting apoptosis including *FN1, GLRX1, CDK2AP1*, and *LGALS3*. Though direct participation of all of these genes has not been evaluated in endometrial cell proliferation or in apoptosis, their role in apoptosis is established. The tumor necrosis factor-α-induced-protein 3 (TNFAIP3) and chemokines including CXCL11 are inducers of epithelial apoptosis [32,33] which is important for species with invasive implantation, however, in pigs, conceptuses do not breach the epithelial layer during implantation. An inhibition in endometrial apoptosis resulting from downregulation of these genes will be favorable for generation of receptive endometrium in pigs. 

Many genes that were upregulated in the endometrium of SP-infused pigs were found to participate in proliferation of microvascular endothelial cells and proliferation of connective tissue cells that consists mostly of fibroblast. The genes associated with these categories included *TGFA*, *MAPK1, ADM, LGALS3, DNMT3B* and *DKK*. Galectins, including LGALS3, are multifunctional proteins associated with immune regulation, cell proliferation, and angiogenesis. Growth factor, TGFA, that activates epidermal growth factor receptor (EGFR) and LGALS3 has been shown to mediate angiogenesis through VEGF and bFGF-mediated angiogenic response [34,35,36]. Increased endometrial angiogenesis is a hallmark of successful pregnancy, it ensures proper growth and development of the embryo. A SP-induced upregulation of these factors suggests its possible effect on endometrial vasculogenesis. 

### 4.3. Oxidation Stress

A downregulation of genes associated with production of reactive oxygen species (ROS) such as *MAOA, NCF2, NOS3* and *CCR3* was observed in endometrial transcriptome of SP-infused pigs. A balance in production ROS is important as at moderate concentrations it has important signaling roles under physiological conditions, but sustained ROS production can have detrimental effects. The expression of *MAOA* which is induced by TNFα has been reported in human and rat endometrium where it is upregulated during the implantation period. The role of this gene in endometrium is not clear yet. However, as *MAOA* is identified as a source of ROS generation, we speculate that a decreased activity of this gene during early pregnancy might create a decrease in detrimental ROS species which in turn might act on downregulation of *CCR3* expression. We also observed an increase in the expression of gene coding antioxidative enzyme, *GLRX*. Glutaredoxins were reported to be expressed in human endometrium where they are associated with the regulation of the cellular redox state and antioxidation defense mechanisms [37]. 

### 4.4. Lipid Metabolism

Processes related to lipid metabolism, such as steroid and lipid quantity and lipid release were found to be inhibited in the endometrium of SP-infused animals (Table S5). Some of the DEGs related to this process included downregulated expression of STAR, NR5A1, and Cyp7A1. Steroid biosynthesis has previously been reported to be regulated by the SP-infusion. However, the process was shown to be activated after 24 h of the SP treatment [25]. In our study, the one probable explanation for downregulation of these genes could be SP-induced upregulation of COX-2 [18]. Though, regulation of steroid biosynthesis by COX-2 has not been evaluated in porcine endometrium, it was shown to be a negative modulator of steroid biosynthesis in Leydig and bovine luteal cells [38,39]. More importantly, it has been suggested that COX-2 inhibits steroidogenesis through MAPK-signaling [40] and MAPK was found to be upregulated after SP-infusion in our study. As we did not measure systemic progesterone concentrations, whether a result of downregulation of these genes was observed in systemic progesterone concentration needs to be evaluated. Interestingly, it has been reported that early progesterone treatment decreases uterine capacity at 105 days of gestation due to accelerated fetal growth [41]. Moreover, a slower rate of conceptus development is attributed to greater fertility as in Meishan breed of pigs. Consistent with these findings, SP has also been shown to increase embryo viability and at the same time slow the growth (size) of embryos on day 9 of gestation [18]. Our observations concerning a possible long-term effect of SP on steroid biosynthesis are worth further exploration in terms of effect of SP on fertility rate in *Sus scrofa domesticus* through moderate changes in progesterone synthesis. 

In conclusion, our results clearly show that SP can induce long term effects on the gene expression in the porcine endometrium. Long-term effects of SP on endometrium include inhibition of processes related to immune response, apoptosis, and steroid biosynthesis and activation of processes such as proliferation of cells. In modern pig breeding, use of SP has been neglected. Our study paves the way for further research on the benefits of addition of SP or its constituents to the semen extenders during AI. The effects of SP on endometrium might prove to be advantageous for blastocyst development, preparing uterus for the conceptus attachment and finally for improving the fertility rate in pigs.

## Figures and Tables

**Table 1:** TaqMan assays used for real-time PCR validation of microarray results.

Gene Symbol	Gene Name	Test ID	Entrez Gene ID
*ACTB* *	*Actin, β*	Ss03376081_ u1	397653
*TGFA*	*Transforming growth factor, α*	Ss03383643_u1	397484
*S100A12*	*S100 calcium binding protein A12*	Ss04246259_g1	100301483
*S100A8*	*S100 calcium binding protein A8*	Ss04246257_g1	100127488
*CCR3*	*Chemokine (C-C motif) receptor 3*	Ss03378176_u1	414373
*CXCL11*	*Chemokine (C-X-C motif) ligand 11*	Ss03648934_m1	100169744
*HPRT* *	*Hypoxanthine phosphoribosyltransferase 1*	Ss03388273_m1	397351
*SLA-DQA1*	*MHC class II histocompatibility antigen SLA-DQA*	Ss03389952_m1	100153387
*IL18*	*Interleukin 18*	Ss03391204_m1	397057
*LGALS1*	*Galectin 1*	Ss03388270_m1	396491
*PDCD10*	*Programmed cell death 10*	Ss03820202_s1	100157978
*LY96*	*Lymphocyte antigen 96*	Ss03389453_m1	100125555

* Reference genes.

**Table 2:** Results of microarray experiment validation with qPCR. 6DP—6 Day of pregnancy vs. 6 Day of estrous cycle, 6DPI—6 Day after SP infusion vs. 6 Day of estrous cycle, Fc—fold change.

Gene Symbol	qPCR		Microarray	
	Fc	*p*-Value	Fc	*p* Corr
6DP				
*IL18*	−3.52	0.002	−3.7	0.036
*LGALS1*	−1.75	0.020	−2.89	0.046
*LY96*	−2.32	0.023	−3.22	0.034
*PDCD10*	−1.85	0.026	−2.16	0.047
*S100A12*	−4.69	0.026	−5.43	0.049
*S100A8*	−5.29	0.014	−9.45	0.041
*SLA-DQA1*	−6.56	0.002	−5.16	0.041
6DPI				
*CCR3*	−6.80	0.011	−8.59	0.003
*CXCL11*	−2.38	0.009	−2.22	0.003
*TGFA*	2.25	0.007	2.64	0.006
*IL18*	−3.64	0.002	−3.54	0.003
*S100A8*	−6.71	0.004	−12.18	0.003
*S100A12*	−6.80	0.011	−5.98	0.002

## Supplementary Materials

The following are available online at https://www.mdpi.com/2073-4425/11/11/1302/s1, Table S1: Microarray analysis data listing differentially regulated genes that were up- or downregulated on day 6 of pregnancy as compared to day 6 of the estrous cycle, Table S2: Microarray analysis data listing differentially regulated genes that were up- or downregulated on day 6 after seminal plasma infusion as compared to day 6 of the estrous cycle, Table S3: List of 19 common differentially regulated genes between day 6 of pregnancy and day 6 after seminal plasma infusion, Table S4: Disease and function analysis (IPA analysis) of DEGs identified on day 6 of pregnancy as compared to day 6 of the estrous cycle, Table S5: Disease and function analysis (IPA analysis) of DEGs identified on day 6 after seminal plasma infusion as compared to day 6 of the estrous cycle, Table S6: List of upstream regulators that might regulate the expression of DEGs identified on day 6 of pregnancy, Table S7: List of upstream regulators that might regulate the expression of DEGs identified on Tabelday 6 after seminal plasma infusion.
