# Supplementary material for: Prolonged Effect of Seminal Plasma on Global Gene Expression in Porcine Endometrium
Source: Genes (Basel). 2020 Nov 3;11(11):1302. doi: 10.3390/genes11111302 (PMC7692128; doi:10.3390/genes11111302)
Supplement: Supplementary file 1 [file genes-11-01302-s001.zip › Supplementary Table 3.docx]

**Supplementary Table 3.** Common DEGs between 6 Day of pregnancy (6DP) and 6 Day after seminal plasma infusion (6DPI) both compared to 6 Day of estrous cycle; *Fc – fold change.

| Gene Symbol | Gene name | Fc* 6DP  vs 6DC | Fc* 6DPI  vs 6DC |
| --- | --- | --- | --- |
| *ATP6V1E2* | *ATPase, H+ transporting, lysosomal 31kDa, V1 subunit E2* | -3,15 | -2,91 |
| *CDKN3* | *Cyclin-dependent kinase inhibitor 3* | -2,29 | -1,55 |
| *CLCN5* | *Chloride channel, voltage-sensitive 5* | -2,66 | 1,53 |
| *COL7A1* | *Collagen,type VII,alpha 1* | 2,40 | 1,96 |
| *CSGALNACT1* | *Chondroitin sulfate N-acetylgalactosaminyltransferase 1* | 4,17 | 3,75 |
| *FKBP3* | *FK506 binding protein 3, 25kDa* | -1,67 | -1,55 |
| *FN1* | *Fibronectin 1* | 2,00 | 1,53 |
| *H2AFZ* | *H2A histone family, member Z* | -1,81 | -1,68 |
| *IL15* | *Interleukin 15* | -2,71 | -2,6 |
| *IL18* | *Interleukin 18* | -3,73 | -3,54 |
| *LGALS1* | *Lectin, galactoside-binding, soluble, 1* | -1,72 | -1,82 |
| *LYSMD3* | *LysM, putative peptidoglycan-binding, domain containing 3* | -1,89 | -1,55 |
| *NDUFAF4* | *NADH dehydrogenase (ubiquinone) complex I, assembly factor 4* | -2,16 | -1,76 |
| *ORMDL1* | *ORM1-like 1* | -1,92 | -1,66 |
| *S100A12* | *S100 calcium binding protein A12* | -9,45 | -5,97 |
| *S100A8* | *S100 calcium binding protein A8* | -9,45 | -12,17 |
| *SNX10* | *Sorting nexin 10 (SNX10)* | -2,1 | -2,8 |
| *SRGN* | *Serglycin* | -3,39 | -3,55 |
| *TUBA1B* | *Tubulin alpha 1b* | -3,32 | -1,54 |
